# Epigenetic Control of MicroRNA Expression and Aging

**DOI:** 10.2174/138920209788185225

**Published:** 2009-05

**Authors:** Ruqiang Liang, David J Bates, Eugenia Wang

**Affiliations:** 1 Gheens Center on Aging, University of Louisville School of Medicine, Louisville, KY, USA; 2 Department of Biochemistry and Molecular Biology, University of Louisville School of Medicine, Louisville, KY, USA

**Keywords:** MicroRNA, promoter, transcription factor, transcription factor binding site.

## Abstract

MicroRNAs are a major category among the noncoding RNA fraction that negatively regulate gene expression at the post-transcriptional level, by either degrading the target messages or inhibiting their translation. MicroRNAs may be referred to as ‘dimmer switches’ of gene expression, because of their ability to repress gene expression without completely silencing it. Whether through up-regulating specific groups of microRNAs to suppress unwanted gene expressions, or by down-regulating other microRNAs whose target genes’ expression is necessary for cellular function, such as cell proliferation, apoptosis, or differentiation, these regulatory RNAs play pivotal roles in a wide variety of cellular processes. The equilibrium between these two groups of microRNA expressions largely determines the function of particular cell types. Our recent results with several model systems show that upon aging, there is a trend of up-regulation of microRNA expression, with concomitant inverse down-regulation of target genes. This review addresses molecular mechanisms that may provide the underlying control for this up-regulating trend, focusing on activation by various microRNAs’ own promoters, through binding with pivotal transcription factors, stress response, methylation of clustered DNA domains, *etc*. Thus, epigenomic control of aging may be due in part to heightened promoter activation of unwanted microRNA expressions, which in turn down-regulate their target gene products. Overriding and dampening the activation of these noncoding RNAs may prove to be a new frontier for future research, to delay aging and extend healthy life-span.

## 1. INTRODUCTION

It has been sixteen years since the first discovery of a minute 22 nucleotide (nt) microRNA (miRNA), lin-4, functionally characterized as regulating a target protein, lin-14, and controlling developmental timing in* Caenorhabditis elegans *[1]. This miRNA/target relationship was later proven to determine nematode life span [2], since lin-14 is an essential transcription factor (TF) with overarching impact upon a wide variety of signaling networks, such as the insulin/insulin-like growth factor pathway [3]. This modest beginning has led to an explosion in identifying not only new miRNAs in *C. elegans*, but also their orthologues in a wide variety of species from viruses to plants, lower animals, and mammals including man; altogether some 8,619 miRNAs are noted and catalogued in the miRBase databases (release 12.0) [4]. It is suggested that the expression levels of more than 60% of human protein-coding genes are controlled by the 695 already identified human miRNAs [5, 6], with more miRNAs yet to be discovered [7].

Of the total 20,000-25,000 protein-coding genes, occupying only 1.2% of the human genome, about six percent are functionally classified as TFs [8]. However, some 93% of our genome is transcribed, by far the greatest part expressed as non-protein-coding RNAs (ncRNA), including the miRNAs [9]. An order of magnitude more numerous than all the proteins which make up living organisms are the transcription start sites (TSSs), located in promoter-proximal element regions, as well as an increasing number of putative promoter-distal elements, identified by the pilot ENCODE project [9].

These recent findings, together with the fact that non-protein-coding genomic sequence elements—such as miRNAs—predominate and are evolutionarily conserved in our genome, challenge our traditional understanding of the definition of a *gene, *which has been generally considered a unit of genome sequence that is transcribed to produce a protein product for a given cellular function. Nevertheless, as the ENCODE consortium suggests, a gene may be defined as “a union of genomic sequences encoding a coherent set of potentially overlapping functional products” that eventually orchestrate the complex regulation and function of the host organism’s cellular activities [10]. An even bolder scenario is proposed by John S. Mattick, who suggests that the genome may consist largely of massively embedded RNA coding sequences directing regulatory networks, which may have co-evolved with proteins. These two complementary genomic sets may ultimately form the interacting RNA-protein regulatory networks which control the complex layers of signaling communication within all cells [11, 12]. Thus, the intriguing notion of epigenomic regulation of essential processes such as cell proliferation, differentiation, apoptosis, *etc*., characterized by feed-forward RNA regulatory networks, is becoming increasingly important in our appreciation of the epigenetic information required for the development of multi-cellular organisms [11]. In this report, we focus our discussion on the suggestion that derailment of the RNA-protein interaction, and its subsequent impact on the regulatory networks which they direct, may constitute a significant fraction of the molecular mechanisms controlling the aging process.

Clearly, multicellular organisms have emerged evolutionarily by means of the specialized functional states of various cell types; both their functions and differentiation lineages require complex regulation of gene expression, and the higher the species, the more complex this regulation [13]. In general, molecular mechanisms regulating gene expression may be determined by inter-related biochemical and molecular processes. These processes include epigenetic regulation [14], *cis*-regulatory elements such as alternative promoters [15], Transcription factor-dependent activation or repression [13, 16], mRNA-dependent actions including splicing, polyadenylation, and finally miRNA-dependent post-transcriptional regulation—including translational inhibition or activation, and mRNA degradation, *etc.* [17-20]. Among the factors involved in these processes, TFs are probably the most important, in activating or repressing the expression of genes *via *target binding to specific *cis*-elements, TF binding sites (TFBSs), which are located proximal to their target’s sequence domain. Many TFBSs are located in clusters called *cis*-regulatory modules (CRMs), that are generally a few hundred base pairs (bp) in size, and may interact with more than a dozen transcription factors (TF). Qualitative and/or quantitative variation of these TFs in a given time frame and specific cell type may be dictated by the CRMs; the TFs and individual modules of CRM interaction produce the temporospatial pattern of expression of the various target genes. Rather than functioning in isolation, TFs may cooperate or interact with each other as co-activators or –repressors; their working partnership may weave the complex networks of signaling required for each space and time within cells [13].

In the last few decades, studies of the regulation of individual gene expressions has been primarily focused at the level of transcriptional control. Now this emphasis is being expanded to the next level of regulation, post-transcriptional regulation by miRNA, which shares the same simple ‘digital’ Watson-Crick sequence complementarity [11], directing miRNAs to post-transcriptionally repress gene expression by specific binding at either 3’-untranslated (UTR) or coding regions [17]. This principle of perfect or imperfect complementary binding results in numerous pair partnerships between individual miRNAs and their targets, giving rise to two molecular scenarios: a single miRNA may target hundreds of messages, and *vice versa* the expression of any message may be repressed by dozens of miRNAs at multiple binding sites [21]. This versatile miRNA/message binding relationship creates an exquisite system of interacting post-transcriptional regulation of gene expression for the complex system-wide signaling networks needed for global programmatic functions in each cell type, while maintaining flexibility for impromptu demands for fine tuning of segmental signaling operations.

This notion of global *versus* segmental signaling controls prompts the suggestion, emanating from computational modeling, that a prevalent mechanism in both the human and mouse genomes is co-regulation of a given miRNA and its many targets. This regulation involves both positive and negative transcriptional regulation of the expression of both molecular partners [22]; within the same signaling pathway, participating miRNAs and their targets function in concert, the two entities together forming miRNA regulatory modules (MRMs). To date 79 such modules are defined in man, from data derived from expression profiles of both miRNAs and their target genes, as well as miRNA-target binding information [23].

As described above, transcription factors (TFs) and miRNAs are two prominent families of regulatory molecules for the control of gene expression, the former functioning at the transcriptional level, and the latter post-transcriptionally. Both species, however, share some functional similarities, such as binding specificity for cell type determination, pleiotropic effect due to the abundance of miRNAs and the availability of a given *cis*-regulatory site for a particular cell state, cooperative and combinatoric effects of multiple miRNAs binding to their targets, and definition of multiple networks by operational motif units. It is becoming increasingly evident that the expression levels of many transcription factors are themselves controlled by miRNAs, such that the expression of miRNAs and their target TFs are linked together in an inverse relationship. In many cases, the miRNA and its targets function in the same signaling pathway, their inverse pattern of expression constituting a sensitive molecular system with feed-back loop operation to fine-tune the amplitude and latitude of cellular signaling [16]. An example of such a relationship is already reported for the miR-200 family and ZEB1/ZEB2 [24-26].

Networks of any type are generally organized in segments of functional units, connected together by ‘hubs’. The bigger the networks, the more single units are connected by ‘hubs’; several such ‘hubs’ may connect together to an upstream ‘hub’. Thus, a larger network may be connected by a few major ‘hubs’, which are then individually linked to dozens of minor ‘hubs’. This scenario of major and minor ‘hubs’ and end-point functional units creates a hierarchical order of network control as exemplified in Fig. (**1**). Evolutionarily, major ‘hubs’ are those most conserved across species from plants to animals, from lower eukaryotes to higher vertebrates, while the minor ‘hubs’ and individual functional units exhibit diversity between species, and provide dynamic, rapid flexibility for a given species’ specialized needs. This hierarchical network scenario is proposed for transcription factor network operation, and probably pertains as well to miRNA functionality, albeit at the post-transcriptional level. Not surprisingly, miRNAs older in the evolutionary time-scale are generally conserved, and their expression is ubiquitous and abundant, while those more recently appearing in the phylogenetic tree are generally less abundant, serving specialized cell or tissue functions [13]. The suggested hierarchical order of ‘hubs’ may be impacted by the range over six orders of magnitude exhibited in the levels of expression of various miRNAs [27]. Questions arising here, including how TFs and miRNAs are co-regulated, and how miRNA expression is regulated by TFs, are emerging as an exciting field of investigation. In this report, we focus on how regulation by miRNA networks impacts post-transcriptionally many signaling processes, determining the molecular mechanisms of aging during the life span of an organism.

## II. REGULATORY NETWORKS OF MICRORNA EXPRESSION

It is by now well defined that most miRNAs are ~22 bases long; since they do not encode any proteins, they are therefore categorically known as small noncoding RNAs (nc-RNA). In general, mature miRNAs are formed by cutting off one arm of a stem-loop precursor miRNA (pre-miRNA), *via *the enzymatic reaction of a cytoplasmic RNaseIII, Dicer. The cytoplasmic presence of pre-miRNAs is due to their release from RNA polymerase (RNAP) II-transcribed long primary miRNAs (pri-miRNA) by a nuclear RNaseIII, Drosha, in a DGCR8 complex in the nucleus; the released pre-miRNA is then transported from the nucleus to the cytoplasm by Exportin5. Functionally, mature miRNAs are complexed together with Argonaute (Ago), the core unit of RNA-induced silencing complexes (RISC) [28]. The repressing of gene expression is accomplished by mature RNA directing the RISC complex to bind to the target messenger RNA, by either perfect complementary binding, for mRNA degradation, or imperfect binding at the 3’UTR region, to inhibit translation; either modality achieves the post-transcriptional suppression of gene expression [17, 28].

Exceptions to this scenario have been noted recently: for example, there is evidence of transcription of a few miRNAs by RNAP III; these miRNAs are separated by interspersed Alu repeats [29, 30**]. In addition, Drosha-independent processing may be seen with pre-miRNAs found in intron regions, termed ‘mirtrons’; they are processed instead by nuclear pre-mRNA splicing [31-33]. There is also another kind of DGCR8-independent endogenous short hairpin-derived miRNA from nonmirtronic genomic loci [33]. These few examples serve to show that many exceptions to the rule exist, and undoubtedly many more new mechanisms remain yet to be discovered.

The production of functional miRNAs, from the initial transcription of the miRNA genes in the nucleus to the generation of mature miRNA in the cytoplasm, may be subject to many levels of regulation [28]. For example, nearly identical mature miRNAs may be generated from different members of the same miRNA gene family; the distinction among members is determined by the pre-miRNA loop nucleotide sequences. Since these sequences are the essential elements for Dicer activity before transforming the pre-miRNAs into mature miRNAs, sister members of a large miRNA gene family may be orchestrated for different required signalings [28]. Thus, the complexities in the biogenesis of miRNA are yet compounded by another layer of regulation, *i.e.* the pre-miRNA stem-loop sequences. In this report, we focus on the regulation of miRNA biogenesis at its first step, regulation by TFs, with the realization that subsequent steps controlling mature miRNA expression are also essential for the putative post-transcriptional regulation of signaling for the aging process.

### miRNA Genomics and Gene Structure

Nearly half of the loci for mammalian miRNAs are located in close proximity to each other, often in a cluster sequence domain, with a ‘master’ promoter for the production of a single polycistronic transcription unit (TU). The remaining miRNA loci may then be structured as individual units, each controlled by its own separate promoter element. Approximately 40% of miRNA loci are located in the intronic region of non-coding transcripts, and ~10% in the exonic region of non-coding TUs; ~40% appear within introns of protein-coding genes. Alternative splicing actions determine whether a miRNA is intronic or exonic, with some miRNA genes located in either region [28]. The mature miRNAs coded by clustered polycistronic transcripts may be either functionally related or distinct [34]. Lengthwise, most intergenic human pri-miRNA genes measure some 3-4 kb, with a distinct 5’- TSS and a CpG island located within the upstream proximal 2 kb region, and also flanked by a poly(A) site within the downstream 2 kb region of the embedded pre-miRNA code. The TFBSs for pri-miRNAs are often found in clusters within the upstream 2 kb region of the pre-miRNAs; the TFs controlling signaling for growth and development are predicted by bioinformatic models to target these clusters [35]. Surprisingly, the length distributions of the two groups of polycistronic and singleton pri-miRNAs are quite similar: half of both groups measure between 1 and 10 kb, most of the remainder of the predicted polycistronic pri-miRNAs are tens of kb long, with a few putative pri-miRNA genes with expanded length up to hundreds of kb. This length distribution for miRNA genes differs from that of protein-coding TUs; in general, the mean length of a protein coding TU for a message is ~50 kb long, and those containing intronic miRNAs are even longer, exceeding 150 kb. As with protein-coding TUs, about half (54%) of the predicted pri-miRNA genes exhibit both promoter and other regulatory sequences, as well as organized exon-intron structural domains, although with fewer intron regions than in protein-coding TUs [36].

### Promoters of MicroRNA Genes

MicroRNA genes are mainly transcribed by RNAPII, and are thus believed to share characteristics and regulation mechanisms of a class II gene. The promoter region of a class II gene is usually separated into three compartments: (1) a ~100 bp long core promoter domain, with TSSs for binding by RNAPII, and binding sites for the general transcription factor (GTF), embedded within it; (2) immediately upstream to this region, a proximal domain several hundred bp long, containing primary specialized regulatory elements; and (3) distal domains, thousands of bp long, constituting secondary regulatory elements [37]. In general, initiation of transcription is activated by RNAPIIs binding to their specific target binding sequence elements within the core promoter. Two different types of core promoters are defined, focused and dispersed; the former contain either a single TSS or a unique cluster of TSSs several nucleotides (nt) in length, while the latter contain several TSSs over 50–100 nt, and are typically embedded in CpG islands in vertebrates. Evolutionarily, focused promoters are more conserved, found in abundance in both invertebrates and vertebrates; however, within the vertebrate kingdom, dispersed promoters are more commonly identified than the focused type. Assemblies of general sequence motifs are often found within the core promoters, including familiar elements such as TATA box, BRE (TFIIB recognition element), Inr (Initiator), MTE (motif ten element), DPE (downstream core promoter element), DCE (downstream core element), and XCPE1 (X core promoter element 1). Combinatoric regulation through the differential binding of these diverse elements supports the versatile control mechanism required for appropriate transcription, response to enhancers, *etc*. [38]. In *silico *predictions suggest that typical TATA-box elements exist in the core promoter regions of a third of all human miRNA genes [39]. Wet lab experimentation combined with bioinformatic studies establish that these motifs are also found within miRNA genes, demonstrating the intricate expression required for various cellular signaling. Similar to known protein-coding genes, about 64% of promoters of miRNAs possess CpG islands within the 500 bp proximal region. Also, TATA elements are found within 19% of miRNA gene promoters, BRE elements in 21%, Inr elements in 47%, MTE in 7%, and DPE in 87%. This description attempts to show that miRNA TSSs identified so far share similar sequence properties with protein-coding genes [30**].

Since most miRNA genes are transcribed by RNAPII, they may be subject to the same regulatory rules as pertain in the transcription of protein-coding TUs. This regulatory modality usually involves the recruitment of RNAPII to the target DNA-binding region, along with various general transcription factors (GTFs), such as TFIIB, TFIID, TFIIE, TFIIF and TFIIH, to form the pre-initiation complex (PIC) which supports binding to specific sites in the promoter domains, enabling subsequent transcription initiation, followed by triage to either promoter escape for abortive transcription, or elongation and finally transcription termination for successful transcription. Integrated in this step are many regulatory factors to facilitate or repress the operation of the PIC at the target binding sites [40]. Key to the entire transcription initiation, recruitment, and formation of co-activator or co-repressor complexes are *cis*-acting sequence domains embedded either in the core promoter regions or in the primary/secondary distal locations, and functionally manifested as either enhancing or repressing elements for transcription [38].

Identification of TF binding properties for the regulation of miRNA transcription has been hampered until recently by dependence upon isolating transient pri-miRNA transcripts from a given cell type, which may or may not contain the entire TF-binding sequence information. A recent approach to solving this problem is the emerging notion that histone modification of DNA is frequently observed at sites of transcription initiation and elongation. In particular, the trimethylation of histone H3 at its lysine 4 residue (H3K4me3) occurs at the TSSs of most genes in the genome; this covalent modification of histone H3 is generally found only at sites of transcription initiation [41]. After mining data generated from many cell types for H3K4me3-enriched loci, Marson *et al*. generated a library of possible TSSs for both human and mouse genomes. By incorporating these signature H3K4me3 sites with the locations of CpG islands and ESTs within the 250 kb upstream regions of individual miRNA genes, they predicted promoter regions in ~1 kb resolution for 294 human pri-miRNAs, which give rise to 441 mature miRNAs [42**].

This strategy of computational biology is validated experimentally by integrating the knowledge that TSSs are in general free of nucleosomes; a 100 to 130 bp window lacking nucleosome binding is usually found to surround the TSSs [43]. Using chromatin immunoprecipitation (ChIP) on chip (ChIP-chip) with one breast cancer and two melanoma lines, Ozsolak *et al*. screened for TSSs within 20 kb upstream and 1kb downstream of all miRNAs, by searching for the binding of H3K4me3, H3K9/14Ac (acetylation of Lys 9/14 of histone 3), RNAPII, and RNAPIII. Their results identify TSSs for 175 human miRNA genes, as well as a short (~70 bp) nucleosome-depleted domain within the core promoter region [30**]. Interestingly, some intronic miRNAs contain TSSs different from those of the host messages in which they are embedded, suggesting that miRNA genes may be transcribed either by their host genes’ TSSs, or independently by their own distinct TSS activation. Variable distances, ranging from a few hundred bp to 20 kb, are found between the start sites of transcription and the coding region of miRNAs [30**].

### Transcription Factors Binding MicroRNA Promoters

Four transcription factors, Oct4, Sox2, Nanog and Tcf3, determine the pluripotent and self-renewing properties of embryonic stem (ES) cells. Interestingly, these four factors occupy 55 unique miRNA TUs in mouse ES cells; the fact that these 55 miRNA TUs give rise to 81 mature miRNAs suggests that about 20% of known mammalian miRNA genes are controlled by these four TFs. The same percentage of protein-coding genes depends on these four factors for their promoter regulation. During differentiation, Oct4 and Nanog are silenced in ES cells, and consequently a significant fraction of their target miRNAs are down-regulated 100- to 1000-fold. A quarter of miRNA genes bound by Oct4/Sox2/Nanog/Tcf3 are not actively transcribed in ES cells, as their promoters are co-occupied by epigenetic regulatory Polycomb group (PcG) proteins, which catalyze H3K27me3 methylation to repress transcription, and thus control specific target gene expressions needed for differentiated cells [42**].

Investigators of the Ozsolak group proceeded from their knowledge of TSSs to investigate the possible existence of well-known transcription elements such as E-box elements within the promoter regions of miRNA genes. Among the many factors binding to E-box elements is the recognized melanoma oncogene, MITF, functionally characterized as the master switch for melanocyte development. Ten E-boxes found in 9 miRNA genes are bound by MITF in a melanoma cell line. Besides MITF, binding to the E-box of miRNA genes is also seen with c-Myc, CREB (cyclic AMP-responsive element binding protein), Suz12 (suppressor of zeste 12 homolog, a PcG protein), and PPAR (peroxisome proliferator-activated receptor) [30**].

Aside from the high-throughput screening approach to identify miRNA promoter regions and the TFs potentially binding to them, many reports take the approach of individual miRNA gene-based investigation. For example, the E-box promoter is found in the 7.9 kb ncRNA transcript regions of miR-199a and miR-214 clusters embedded in the Dynamin-3 gene intron domain [44]. Functionally, aside from its pro-proliferation properties, c-Myc suppresses 13 miRNA genes controlled by 21 unique TUs [45]; c-Myc up-regulation is also associated with the pro-tumorigenic nature of the miR-17-92 cluster [46]. Several groups have shown that members of the miR-34 family are directly controlled by p53, which induces apoptosis, cell cycle arrest, and senescence [47].

### Predominant Up-Regulation of MicroRNAs During Aging

By array profiling of global miRNA expression, our results show more miRNAs up-regulated than down-regulated during aging. miRNA expression levels were profiled in the livers of 4, 10, 12, 18, and 33 month old C57/Black mice. Among the up-regulated miRNAs identified, the levels of miR-669c and -709 start to increase at 18 months, the mid-life of the mouse lifespan, and reach maximal levels at 33 months. This trend is not observed for miR-93 and -214, which show significant up-regulation only at 33 months, extreme old age for mice; we identified very few miRNAs significantly down-regulated with age [48].

Why are more miRNAs up-regulated with age, and what are the underlying mechanisms controlling this up-regulation? We suggest that the main factor may be aberrant control of TFs binding at the promoter region of key up-regulated miRNAs. For example, the transcription start site for mmu-669c was first identified by Marson, *et al*. 2008, and reported in their supplementary table [42**]. The gene for mmu-miR-669c is located in one intron of the gene, Sfmbt2 (Scm-like with four mbt domains 2), a PcG protein, functionally known to interact with transcription factor Yinyang1 (YY1), possibly forming a PcG silencing complex. Embedded in this intron is a large cluster of more than a dozen microRNAs, including miR-669c [49]. Three CpG islands reside in the proximal region of mmu-miR-669c; the first contains a candidate TSS, 137 kb upstream from mmu-669c [49]. This embedding of a large cluster of miRNAs with their own transcription unit is feasible because the host gene, sfmbt2, is extremely long, 225 kb, but its own transcripts only take up 7.7 kb, leaving plenty of space for the miRNA gene cluster [49]. The gene for the other miRNA showing mid-life up-regulation, mmu-miR-709, is found in intron 8 of transcription factor Rfx1 (regulatory factor for X-box 1); the TSS is located in a CpG island. Functionally, Rfx1 is involved as a TF in the activation of gene expression for viruses, including human hepatitis B virus and cytomegalovirus, while suppressing cellular genes such as c-myc and the proliferating cell nuclear antigen. Rfx1 also mediates the serum-induced immediate early response of Id2 (inhibitor of DNA binding/differentiation) expression, by binding to the TSS of Id2 [50]. Mmu-miR-709 also targets the protein encoded by a gene called Brother of the Regulator of Imprinted Sites (BORIS), an important regulator of DNA methylation and imprinting. Tamminga *et al*. showed that in testis, X-ray irradiation causes DNA damage-induced, and Rfx1-mediated, increase of miR-709 expression; this may function as a protective mechanism to reduce the level of BORIS in cells, so as to prevent massive aberrant DNA methylation, *etc*. [51].

### Stress-Responsive miRNAs

Perhaps the accumulation of stress and its deleterious consequences is the most popular theory to explain age-dependent decline [52]. Not surprisingly, miRNAs as the post-transcriptional control for gene expression are intrinsically linked to the functional decline, and the aberrant system homeostasis, during aging. Stress-induced impact on miRNA biogenesis may induce a cascade of downstream molecular and biochemical aberrancy, precipitating the phenotypic manifestations seen in aging organisms. Among the many steps involved in the biogenesis of mature miRNAs, Dicer is exquisitely sensitive to these insults; its function is inhibited by stresses including reactive oxygen radicals, exposure to phorbol esters, and action of Ras oncogene [53]. In HeLa cells, TNFα (tumor necrosis factor α)-induced rapid apoptotic death is accompanied by the down-regulation of Dicer, suggesting the apoptosis-associated cellular insults impacting on the decreased level of this enzyme, which in turn may affect the biogenesis of mature miRNAs [54].

MicroRNA-210 and -373, induced as a cellular response to hypoxic stress, function to dampen the level of key proteins involved in DNA repair, and the observed genetic instability in cancer cells [55]. To date, the best studied stress-responsive factor for miRNA expression is the transcription factor hypoxia-inducible factor-1α, (HIF-1α), known to target the miR-17-92 cluster [56]. HIF-1α also activates miR-210 [57]; in human breast cancer cells, over-expression of hsa-miR-210 is induced by hypoxia, dependent on the function of HIF-1α and VHL (von Hippel-Lindau syndrome protein, involved in the ubiquitylation and degradation of HIF) [58]. A substantial number of miRNAs are up-regulated by exposure to low oxygen environment, including miR-23, -24, -26, -27, -103, -107, -181, -210, and -213; some of these up-regulations are HIF-dependent. In addition, within this group, miR-26, -107, and -210 decrease pro-apoptotic signaling upon exposure to hypoxic environment; thus these miRNAs may indirectly affect tumor formation, by allowing cancerous cells to survive. Not surprisingly, a significant number of hypoxia-induced miRNAs are also over-expressed in many human tumors [59].

Known for years as the ‘guardian of the genome’, p53 is activated in response to assorted insults including DNA damage, depletion of dNTP, low-oxygen hypoxia conditions, and even the effect of oncogenes. Up-regulation of p53 is induced indirectly by miR-29 family members; this is due to the fact that this microRNA represses the negative regulators of p53, p85α and CDC42 [60]. p53 also activates the expression of the miR-34 family, which in turn suppresses the cyclin-dependent kinases required for cell cycle traverse [47]. Halting cell cycling at either G_1_ or G_2_ checkpoints is also associated with genotoxic, stress-dependent p53 activation, up-regulating homologous tumor suppressor miR-192/215, which functionally targets gene expressions needed for the traverse of these two checkpoints of cell cycle traverse [61]. Other targets of miR-34, including SIRT1 protein, *etc*., are described in detail in the chapter in this issue by Apana Takwi and Yong Li titled “The p53 pathway encounters the microRNA world”.

Hypertrophy of adult cardiomyocytes may be induced by many forms of injury and stress, perhaps due to over-expression of several stress-responsive miRNAs, including miR-23a, -23b, -24, -195, and -214. Likewise, specific over-expression of miR-195 induces cardiac hypertrophy to the extent of causing heart failure in transgenic mice [62]. Within intron 27 of α-myosin heavy chain (αMHC) is embedded cardiac-specific miR-208, linked to cardiomyocyte fibrosis and hypertrophy, and expression of βMHC. Mice deficient for miR-208 are short-lived after birth, due to progressive heart failure and inappropriate up-regulation of fast skeletal muscle and stress response genes [63].

Another stress-responsive TF/miRNA gene pair may be NF-κB and hsa-miR-146a. In Alzheimer’s disease brain, up-regulation of has-miR-146a is found in inverse relationship with the down-regulation of its target gene, complement factor H (CFH), which is an important repressor of inflammatory response in the brain. In primary cultures of oxidatively stressed human neural cells, treatment with interleukin-1β and/or Aβ42 increases the expression of NF-κB. The activation of miR-146a is controlled by NF-κB through binding to three different target domains of this miRNA’s promoter [64]. Since miR-146a also targets TNF receptor-associated factor 6 (TRAF6) and IL-1 receptor associated kinase (IRAK1), and since both of these can activate downstream NF-κB, this forms yet another feed-back loop between a TF and miRNA gene expression [65]. Thus, NF-κB activates the production of miR-146a, which suppresses the TRAF6 and IRAK1 required for its activation, an intricate mechanism ensuring the balanced expression of NF-κB at the desired level in cells.

One of the most consistently observed declines encountered during aging is stress attenuation: the robustness of the response to stress is diminishing in aged animals. Therefore, maintaining reduced levels of stress-responsive gene expression allows an ample reservoir to respond to sudden needs. Hsa-miR-20a, -93, -106b, -373, and -520d repress the expression of MICA and MICB, functioning as stress-activated ligands recognized by receptor NKG2D; the ligand and receptor are involved in the removal of tumor cells and invading virus particles. Since exposure to many insults, including heat shock, oxidative stress, viral infection, and DNA damage, induce up-regulated MICA and MICB expression, constitutive repression of these two proteins’ abundance to lower levels should permit their rapid acute activation when needed. Thus, the function of these miRNAs may be viewed as defending stress attenuation. However, excessive over-expression of these miRNAs, as in neoplasia, induces the inability to form the NKG2D complex needed for the removal of the tumor cells [66]. Again, this suggests the need to have miRNA expressions balanced at the proper threshold levels, allowing the avoidance of stress attenuation without the loss of tumor removal capability in the immune system.

### Epigenetic Regulation of miRNA Expression

Among the many biochemical controls of epigenetic impact in gene expression, DNA methylation may be the most dominant. Since DNA methylation is intimately involved with histone modification, it is not unreasonable to suggest that the post-translational modification of histone and its associated partners may also be vital factors governing the success of transcriptional outputs needed to achieve the cellular signaling required for many biological events, such as cell cycle traverse, apoptosis, response to environmental insults, *etc*. [14].

Most DNA modification involves covalent modification by methylation at the cytosine bases of CpG dinucleotides, the backbones of CpG islands. Hypermethylating the CpG islands in the promoters of miRNAs suppresses their transcription, perhaps by interacting with methyl-CpG-binding proteins (MeCPs). In addition, DNA methylation in mammalian cells is largely dictated by three major protein components, Dnmt1, Dnmt3A, and Dnmt3B, of the DNA methyltransferase (DNMTs) family [14]. Obviously, methylation of miRNA promoters may be pivotal to determining the ability of miRNA transcripts to be successfully produced, and dys-regulation of this biochemical event may induce the loss of essential miRNAs, and subsequent pathological developments in tissues. This scenario is mostly noted in cancer. Aberrant hypermethylation is found in the genes of mir-9-1, -124a3, -148a, -152, and -663 in many human breast cancer tissues [67]. Treating lymph node-derived metastatic cancer cells with inhibitors of DNA methylation such as 5-aza-2’-deoxycytidine induces up-regulation of the expression of 57 miRNAs, among which 27 bear characteristic CpG islands in their promoter regions; 16 of these 27 exhibit hypermethylated CpG islands, typical of cancers, while the remaining 11 miRNAs’ CpG islands are equally hypermethylated in cancerous and normal cells. Notwithstanding the importance of methylation at CpG islands, miRNA-directed post-transcriptional regulation of other factors such as the three methyltransferases [68, 69] may be indirectly linked to the methylation status of miRNA promoters, and therefore their activation. Clearly, methylation at CpG islands of miRNA promoters is a fertile field of research for the study of aging; future work should elucidate whether age-dependent qualitative and/or quantitative impact of this biochemical event precipitates the up-regulation of specific miRNAs in aged animals.

## III. CONCLUSION AND PERSPECTIVE

Our recent findings from several aging model systems can be summarized as: 1. during aging, predominantly up-regulated miRNA expression is observed; 2. in liver, this up-regulated trend emerges at 18 months, the mid-life of a mouse’s life span; and 3. parallel proteomic profiling and bioinformatic mapping show that the up-regulated miRNA expression corresponds to the down-regulation of genes functionally involved in the control of intermediate metabolism, apoptosis, DNA repair, oxidative defense, particularly mitochondrial oxidative phosphorylation, *etc*. In addition, our work with cultured fibroblasts shows that stress, such as peroxide treatment, can induce the trend of up-regulating miRNA expression. In particular, permanent growth arrest, seen either in replicative senescence *via *serial passaging, or in premature senescence induced by peroxide treatment, is associated with significant up-regulated expression of miRNAs. These culture models suggest that in stress-dependent activation of miRNA up-regulation, as in tissues during aging, accumulation of stress may be a major factor inducing the same up-regulating trend.

**Fig. (2):**
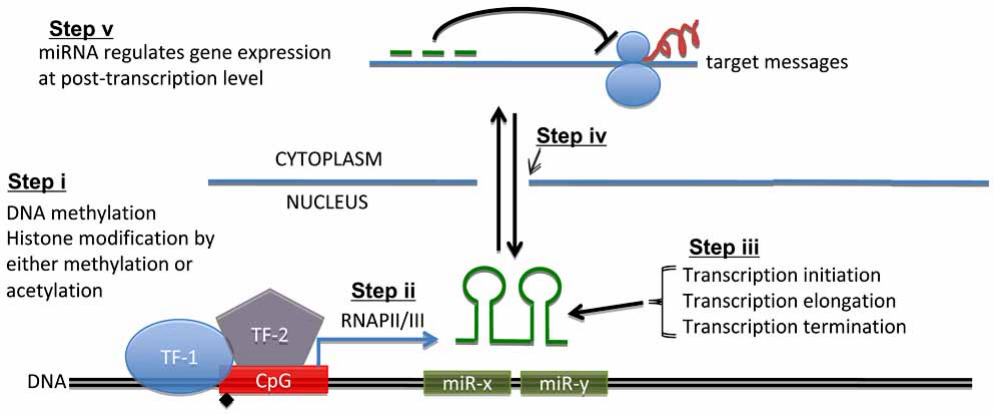
**Suggested model for the regulation of miRNA expression.** MicroRNA expression may be regulated at both transcriptional and post-transcriptional levels. **Step i:** transcription activation or repression by DNA and/or histone modification, for binding by transcription factors (TFs) for either activating or repressing the promoter elements of the microRNA gene clusters; **Step ii:** transcription of miRNA gene by RNA polymerase II (RNAP II) and/or RNA polymerase III (RNAP III); **Step iii:** putative factors regulating RNAP functions for initiation, elongation, and termination steps of microRNA gene clusters; **Step iv:** export of the primary transcripts of miRNA (pri-miRNA), followed by further processing to become precursor microRNA (pre-miRNA), which are exported from the nucleus to the cytoplasm; **Step v:** in the cytoplasm, pre-miRNAs are processed further to mature miRNAs, which can then silence the target message’s expression.

Clearly, emerging from the above description is the obvious question, why and how during aging is miRNA expression up-regulated? The answers may lie in at least three areas, *i.e.* transcription factors (TFs), stress response, DNA methylation, *etc.* (Fig. **2**)*.* TFs have long been known to be essential to the expression of key genes which accelerate or delay the aging process. Interestingly, these TFs may themselves functionally regulate the very miRNAs that target their expression, thus forming feed-back loops between the two molecular categories. Moreover, the acetylation status of these factors may introduce another layer of control for their function. Thus, dysregulation of the balanced expression between miRNAs and their target TFs may then be the very molecular force driving the disequilibrium of many cellular processes seen during aging. Notwithstanding the importance of miRNA/TF loops in controlling the aging process, the fact that most miRNA genes are found in clusters, and a single promoter element can control both the proximal and distal sequence domains of these ncRNAs, introduces another layer of complexity, *i.e*. the DNA methylation and histone modification of these genomic domains, dictating the folding and unfolding of the necessary structural features for gene expression. Moreover, the stability and repair of promoter DNA structures emerge as essential up-stream factors determining the proper expression of miRNAs. Stress-induced damage in these regions, to either genomic DNA or the proteins that bind to them, may equally impact promoter regulation, thus inducing deleterious miRNA up-regulation during aging. In all, DNA methylation, histone modification, and DNA repair are essential biochemical processes, necessary to maintain proper patterns of miRNA expression; factors involved in these three processes, thus controlling miRNA expression, along with the miRNAs themselves, have evolved as ‘Epigenomic Regulators of the Aging Process’. Future research in this area should yield unprecedented new insights as to how the aging process is controlled epigenomically.

## Figures and Tables

**Fig. (1):**
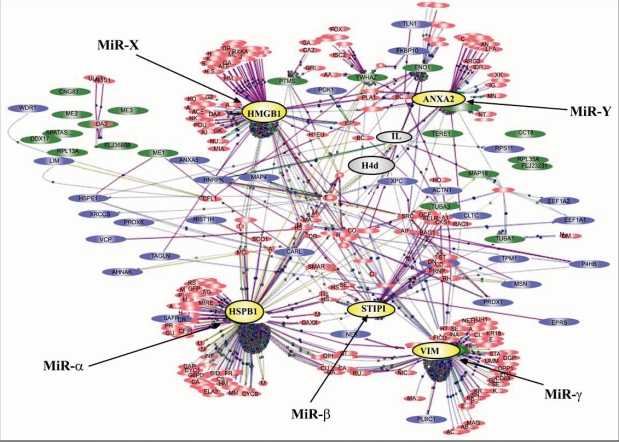
**An example of a signaling network for peroxide-induced premature senescence in human fibroblasts, demonstrating major and minor ‘hubs’; the former are possible target sites of five different putative microRNAs.** This network was constructed by the Ingenuity functional clustering software, with lead proteins identified as differing in concentration between young replicating WI-38 human fibroblasts and their senescent counterparts exposed to peroxide, and their prematurely senescent counterparts (induced by peroxide). The five major hubs, represented here as yellow color-filled circles, are: High-mobility group box 1 (HMGB1), Annexin A2 (ANXA2), Vimentin (Vim), Heat-shock protein B1 (HSPB1 or Hsp27), and Stress-induced phosphoprotein (STIP1); and the two minor hubs, represented here as two gray color-filled circles, are histone 1 (H4d) and interleukin (IL). In this model each of the five major hubs is targeted by one of five putative microRNAs, i.e. miR-X, miR-Y, miR-α, miR-β, and miR-γ. However, not shown here are the two potential scenarios that: 1. an individual ‘hub’ may be targeted by multiple microRNAs; and 2. miR-X may target not only HMGB1 but also HSPB1, according to the multi-miR/multi-target pair principle.
